# Drug sales data analysis for outbreak detection of infectious diseases: a systematic literature review

**DOI:** 10.1186/s12879-014-0604-2

**Published:** 2014-11-18

**Authors:** Mathilde Pivette, Judith E Mueller, Pascal Crépey, Avner Bar-Hen

**Affiliations:** EHESP French School of Public Health, Sorbonne Paris Cité, Rennes, France; Université Paris Descartes, MAP5, Paris, France; Celtipharm, Vannes, France; Institut Pasteur, Emerging Diseases Epidemiology Unit, Paris, France; Aix Marseille Université, IRD French Institute of Research for Development, EHESP French School of Public Health, UMR_D 190 “Emergence des Pathologies Virales”, Marseille, France

**Keywords:** Disease outbreaks, Syndromic surveillance, Drug utilization, Nonprescription drugs, Communicable diseases, Epidemiology

## Abstract

**Background:**

This systematic literature review aimed to summarize evidence for the added value of drug sales data analysis for the surveillance of infectious diseases.

**Methods:**

A search for relevant publications was conducted in Pubmed, Embase, Scopus, Cochrane Library, African Index Medicus and Lilacs databases. Retrieved studies were evaluated in terms of objectives, diseases studied, data sources, methodologies and performance for real-time surveillance. Most studies compared drug sales data to reference surveillance data using correlation measurements or indicators of outbreak detection performance (sensitivity, specificity, timeliness of the detection).

**Results:**

We screened 3266 articles and included 27 in the review. Most studies focused on acute respiratory and gastroenteritis infections. Nineteen studies retrospectively compared drug sales data to reference clinical data, and significant correlations were observed in 17 of them. Four studies found that over-the-counter drug sales preceded clinical data in terms of incidence increase. Five studies developed and evaluated statistical algorithms for selecting drug groups to monitor specific diseases. Another three studies developed models to predict incidence increase from drug sales.

**Conclusions:**

Drug sales data analyses appear to be a useful tool for surveillance of gastrointestinal and respiratory disease, and OTC drugs have the potential for early outbreak detection. Their utility remains to be investigated for other diseases, in particular those poorly surveyed.

**Electronic supplementary material:**

The online version of this article (doi:10.1186/s12879-014-0604-2) contains supplementary material, which is available to authorized users.

## Background

Since the mid-1990s and the raise of concerns about bioterrorism and emerging diseases, non-diagnosis-based data have increasingly been used for routine disease surveillance and outbreak detection [1]. The CDC defined “syndromic surveillance” as an investigational approach where health department staff, assisted by automated data acquisition and generation of statistical alerts, monitor disease indicators in real-time or near real-time to detect outbreaks of disease earlier than would otherwise be possible with traditional public health methods [2].

In such efforts, different registries have served as data sources for public health surveillance [1],[3], including data on absenteeism at work or school [4], calls to health helplines [5],[6], emergency department consultations [7],[8], ambulance dispatching [9], or drug sales. Although unspecific, such data sources can have the advantage over diagnosis-based surveillance of providing information within short delays since the event and in readily available electronic form for relatively low-cost, while capturing large parts of the population.

Drug sales data analysis may overcome the limitation of poor specificity when groups of drugs are exclusively used for the disease or disease syndrome of interest. Furthermore, drug sales data may earlier capture changing population health status, as over-the-counter (OTC) sales and a dense network of pharmacies in most developed countries make drugs easily accessible to patients at the earliest appearance of their symptoms.

Despite this potential interest, no state of the art of drug sales based surveillance is available to date. The present systematic literature review therefore summarized the evidence for an added value of drug sales data for infectious disease surveillance. We limited the scope of the review to infectious diseases, as they represent a public health problem for which early and valid signal detection is of particular concern, in light of potentially rapid emergence and opportunity for control interventions.

## Methods

We conducted a literature search from 1975 up to June 2012 to identify relevant peer-reviewed articles regarding surveillance of infectious diseases based on drug sales data. PRISMA guidelines were followed in the reporting of the review [10]. Published articles were searched for on electronic databases (Pubmed, Embase, Scopus, LILACS, African Index Medicus, Cochrane Library), using combinations of the following key words: (“surveillance” OR outbreak detection OR warning system) AND (over-the-counter OR “prescription drugs” OR pharmacy OR (pharmaceutical OR drug OR medication) sales). The search was limited to articles in English or French. There were no limitations on study settings.

To be included in the review, articles had to describe, test, or review an infectious disease surveillance based on drug sales data; and be original research that presented new data and results. We excluded studies that monitored chronic diseases, as well as prevalence studies whose purpose was not epidemic detection.

One reviewer screened and evaluated the titles and abstracts. Articles were widely included in a first stage. The full-text review and the final selection of the articles were made by two reviewers.

We reviewed and described the articles in terms of objectives, diseases studied, data sources, methodologies, and performance for real time surveillance. To describe methods and results, we separated the articles into three groups based on their main objective: descriptive retrospective studies, drug selection studies, and prediction studies. Outcomes selected to compare drug sales data to reference surveillance data of the corresponding disease were correlation measurements (strength and timeliness of the correlation) and indicators of outbreak detection performance (sensitivity, i.e. ability to identify true outbreaks; specificity, i.e. ability to identify true negative and timeliness of the detection).

## Results

**Figure 1:**
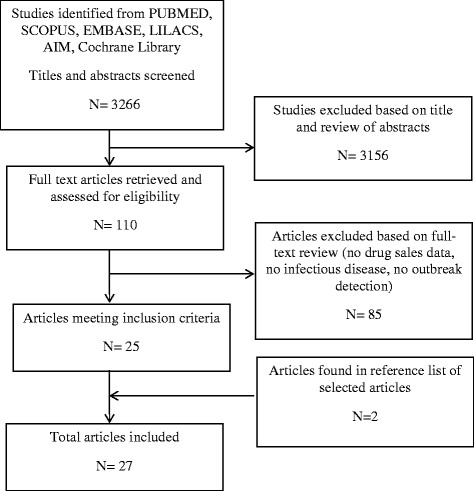
**Flow chart of study selection process in a systematic review of drug sales data analysis for syndromic surveillance of infectious diseases.**

We screened a total of 3266 articles, of which 27 were included in the final review. The search and selection process is presented in Figure 1.

### Objectives of the studies

Three types of studies were defined: retrospective descriptive studies, drug selection studies and prediction studies.

Nineteen of the 27 studies were descriptive retrospective studies assessing the strength of the correlation between drug sales and reference surveillance data of the corresponding disease or evaluating outbreak-detection performance [11]-[30]. Five studies used statistical algorithms to select groups of drugs that were closely associated with clinical surveillance data of a given disease and that would be most appropriate for future drug-sales-based surveillance [21],[31]-[34]. In a third group of three studies, the authors developed and evaluated statistical models to predict clinical surveillance data based on drug sales data [35]-[37]. Table 1 summarizes the studies in terms of their general characteristics.

**Table 1:** **General characteristics of studies included in a literature review of drug sales data analyses for surveillance of infectious diseases**

Author	Study period	Location	Syndrome	Drug data sources	Drug status	Time scale
**RETROSPECTIVE STUDIES**						
Sugawara et al. [27] 2012	2009/2010	Japan	ILI	Prescription drug purchases from 5275 pharmacies	Prescribed	Week
	2010/2011					
Polgreen et al. [23] 2011	Jan 1999– Sept 2007	Ohio, USA	GI	IMS Health’s Xponent database (Data from retail pharmacies, covering 70% of all prescriptions)	Prescribed	Month
Chopra et al. [12] 2011	April–Dec 2009	Southeastern Michigan, USA	ILI	In-site pharmacies of eight hospitals in Southeastern Michigan	Prescribed	Week
Kirian et al. [19] 2010	July 2003-Dec 2007	San Francisco Bay area	GI	Sales available through the National Retail Data Monitor in three San Francisco Bay Area counties (number of stores reporting per week: 592-837)	OTC	Week
Yoshida et al. [30] 2009	Dec 2006-April 2007	Sahai City, Osaka, Japan	ILI	Questionnaires mailed to 273 pharmacies. 56% responding pharmacies. (456,000 people)	Prescribed	Week
Van den Wijngaard et al. [28] 2008	2001-2003	The Netherlands	ILI	Foundation for pharmaceutical statistics (85% of Dutch pharmacies) (13,8 million people)	Prescribed	Week
Edge et al. [17] 2006	Jan 2001-April 2004	Canada (one province)	GI	One major retailer with 19 locations (12% of the pharmacies in the region)	OTC	Week
Das et al. [14] 2005	Aug 2002-March 2005	New York City	ILI and GI	New York City Department of Health (248 NYC pharmacies, 30% of citywide sales)	OTC	Day
Okhusa et al. [22] 2005	Nov 2003-April 2004	Japan	ILI	Private marketing company (1100 pharmacies, 2% of the pharmacies in Japan)	OTC	Day
Chen et al. [11] 2005	March 2003-July 2004	A rural county in New York State	Pertussis	Medicaid database reimbursement	Prescribed	Day
Edge et al. [16] 2004	Jan-May 2001 (Saskatchewan) March-June 2000 (Ontario)	Battleford (Saskatchewan), Walkerton(Ontario)	GI	One pharmacy in Battleford, one pharmacy in Walkerton	OTC	Week
Magruder et al. [21] 2004	2001-2003	Maryland-Washington-Virginia	ILI	Johns Hopkins Applied Physics laboratory (300 drugstores in the Maryland-Washington-Virginia area)	OTC	Day
Couturier et al. [13] 2004	2001-2003	Paris and five french regions	Syphilis	A centralized wholesaler supplying all French private pharmacies	Prescribed	Month
Hogan et al. [18] 2003	1998-2001	Pennsylvania, Indiana, Utah	ILI and GI	Information Resources, Inc., private company (90% market share in all the region)	OTC	Week
Magruder [20] 2003	2001-2002	Maryland-Washington-Virginia	ILI	Johns Hopkins APL (300 drugstores in the Maryland-Washington-Virginia area)	OTC	Day
Davies et al. [15] 2003	1998/1999, 1999/2000, 2000/2001	Nottingham city	ILI	Retailers (Boots the chemist, 30% of market share) in the hospital area, and a pharmaceutical company (Reckitt Benckiser) at national level	OTC	Week
Stirling et al. [26] 2001	Jan-May 2001	North Battleford, Saskatchewan	GI	Three pharmacies	OTC	Week
Proctor et al. [24] 1998	1993	Milwaukee (Wisconsin, USA)	GI	One pharmacy	OTC	Day
Rodman et al. [25] 1997	1993(Milkauwee)/1994 (Las Vegas)/1996 (Collingwood,Kelowna, Cranbrook)	Milwaukee (Wisconsin); Collingwood (Ontario), Kelowna, Cranbrook (British Colombia), Las Vegas(Nevada)	GI	One pharmacy in Milwaukee, 3 pharmacies in Collingwood, 10 to 12 pharmacies in Kelowna and Cranbrook.	OTC	Month
Welliver et al. [29] 1979	1976/1977	Los Angeles	ILI	One large supermarket chain	OTC	Week
**DRUG SELECTION STUDIES**						
Pelat et al. [33] 2010	2000-2009	France	GI	IMS Health (7,500 pharmacies in 2000-13,300 in 2008, 59% of the pharmacies in France, covering all continental France)	OTC and prescribed	Week
Cami et al. [31] 2009	Jul 2003- Dec 2006	Region around the city of Houston, TX, USA	ILI	AC Nielsen Corp (pharmacy sales in a region around Houston)	OTC	Week
Wallstrom et al. [34] 2007	2002-2004	Pennsylvania, USA	ILI	AC Nielsen Corp (pharmacy sales in western Pennsylvania)	OTC	Week
Li et al. [32] 2005	Jan 1998- Dec 2001	Pennsylvania, USA	ILI and GI	Information Resources, Inc., private company (90% market share in the region)	OTC	Week
Magruder et al. [21] 2004	2001-2003	Maryland-Washington-Virginia	ILI	Johns Hopkins APL (300 drugstores in the Maryland-Washington-Virginia area)	OTC	Day
**PREDICTION STUDIES**						
Vergu et al. [37] 2006	2000-2004	France	ILI	IMS Health (11,000 pharmacies, 50% of all pharmacies in France)	OTC and prescribed	Week
Najmi et al. [36] 2005	2001-2003	Maryland-Washington-Virginia	ILI	Johns Hopkins APL (300 drugstores in the Maryland-Washington-Virginia area)	OTC	Day
Najmi et al. [35] 2004	2001-2002	Maryland-Washington-Virginia	ILI	Johns Hopkins APL (300 drugstores in the Maryland-Washington-Virginia area)	OTC	Day

*Abbreviations: ILI* Influenza-like illness, *GI* Gastrointestinal, *OTC* Over-the-counter drugs.

### Diseases studied

Most of the studies focused on respiratory illnesses (17 studies) [12],[14],[15],[18],[20]-[22],[27]-[32],[34]-[37] or gastrointestinal illnesses (11 studies) [14],[16]-[19],[23]-[26],[32],[33]. Only two other studies evaluated surveillance of pertussis [11] and syphilis [13].

### Populations surveyed

Most of the studies were set in the United States (*n* = 16 studies, 59%), followed by Canada (*n* = 4), France (*n* = 3), Japan (*n* = 3), the Netherlands (*n* = 1) and England (*n* = 1). Only one study was conducted in more than one country [25].

### Drug sales data sources

In most retrospective studies, data were collected specifically for the purpose of the study from a sample of pharmacies [16],[24]-[26] or from retailers [13],[15],[17],[29]. For example, in a Canadian study [17], electronic data were provided by one major retailer for all of their 19 pharmacies in the study area.

Automatically compiled data sources were used in all the drug selection and prediction studies and in some retrospective studies. Drug sales data were routinely collected in samples of a city’s or country’s pharmacies. Such routine data collection systems were mainly implemented by research or public health groups, such as the Johns Hopkins Applied Physics Laboratory [20],[21],[35],[36], the New York City Department of Health [14], the National Institute of Infectious Diseases in Japan [27], or the Real-Time Outbreak and Disease Surveillance Laboratory at the University of Pittsburg [19]. Data are available the day after the day’s sales in those systems. In eight other studies, private marketing companies had automatically aggregated and made available drug sales data from a sample (2-90%) of pharmacies in a given city or country [18],[22],[23],[31]-[34],[37].

### Methods and results by study types

#### Retrospective studies

Nineteen studies retrospectively compared drug sales data to gold standard reference data of the disease. Details are given in Table 2.

**Table 2:** **Methodology and results of the retrospective studies included in a literature review of drug sales data analyses for surveillance of infectious diseases**

Author	Syndrome	Reference data sources	Drugs selected	Statistical methods	Correlation strength	Correlation timeliness	Detection sensitivity	Detection specificity	Detection timeliness
**STUDIES ON PRESCRIBED DRUGS**									
Sugawara et al. [27] 2012	ILI	Influenza cases from 5000 hospitals and clinics	Drugs against influenza virus : oseltamivir, zanamivir, laninamivir	Correlation	Pearson corr. coeff. r = 0.992 for 2009/10 and r = 0.972 for 2010/11 (*p*<0.001)	-	-	-	-
Polgreen et al. [23] 2011	GI	Hospitalizations with diagnosis of Clostridium Difficile Infections	Oral vancomycin	Cross-correlation, Regression model	Increase in Clostridium Difficile Infections associated with increase in vancomycin use	-	-	-	-
Chopra et al. [12] 2011	ILI	Cases of influenza reported from nine sentinel healthcare providers	Oseltamivir	Correlation	Spearman corr.coeff. r = 0.46 (*p*<0.003) Peaks occurred at the same time	-	-	-	-
Yoshida et al. [30] 2009	ILI	28 sentinel surveillance sites of influenza in Sahai City (clinics and hospitals)	Oseltamivir and Zanamivir	Correlation	Pearson corr.coeff. r = 0.954	-	-	-	-
Van den Wijngaard et al. [28] 2008	ILI	Respiratory pathogen diagnosis in laboratory registries (Influenza A, B, RSV, enterovirus, S.pneumoniae..)	Drugs for respiratory infectious diseases (7 ATC classes)	Graphical comparison, Correlation, Linear regression model	Pearson corr .coeff. r = 0.60 for Influenza A, r = 0.58 for RSV, r = 0.60 for S. pneumonia, r = 0.39 for influenza B (p<0.05) 80% of variation explained by respiratory pathogens	2 weeks earlier until 1 week later	-	-	-
**Author**	**Syndrome**	**Reference data sources**	**Drugs selected**	**Statistical methods**	**Correlation strength**	**Correlation timeliness**	**Detection sensitivity**	**Detection specificity**	**Detection timeliness**
Chen et al. [11] 2005	Pertussis	Reported cases of pertussis from the NYS department of Health	Macrolide antibiotics	CUSUM	-	-	100% The signal was indicator of pertussis outbreak	100%	Not early warning
Couturier et al. [13] 2004	Syphilis	Reported cases of syphilis from hospitals, physicians, sexually transmitted disease clinics.	Benzylpenicillin benzathine 2.4 MUI	Descriptive analysis	Similar trend (+22% increase in Paris, +10% in the 5 regions)	Similar trend	-	-	-
**STUDIES ON OTC DRUGS**									
Kirian et al. [19] 2010	GI	Cases of gastrointestinal diseases from County Health Department and detected GI outbreaks	Diarrheal remedies (based on common use)	Cross-correlation, Regression ARIMA	No significant correlation between sales and GI cases counts, outbreak counts.	-	Not sensitive 4%-14%	Specific 97%-100%	-
Edge et al. [17] 2006	GI	Counts of GI cases due to bacteria, parasites, and viruses	Antinauseant and antidiarrheal products	Cross-correlation	Temporal patterns of OTC and Norovirus activity were similar Pearson r^2^ = 0.44	No delay	-	-	-
Okhusa et al. [22] 2005	ILI	Reporting of patients with ILI (hospitals, clinics, physicians)	Most common treatments ILI	Cross-correlation, Prediction model, Peak comparison	Significant correlation between sales and influenza activity	Sales do not determine influenza in advance	-	-	-
Das et al. [14] 2005	ILI	Emergency department in New York City (ratio of ILI syndrome visits/other syndrome visits)	A cold medication selected statistically from a group of 400 cold medications (ratio ILI/analgesics drugs sales)	Cross-correlation, Serfling method, Graphical comparison	High correlation Pearson r^2^ = 0.60 (*p*<0.001)	No lead time	Sensitive (data not reported)	Not specific (not reported)	Not earlier warning than reference data
Das et al. [14] 2005	GI	Emergency department in New York City (ratio of GI syndrome visits/other syndrome visits)	Common antidiarrheal drugs(ratio GI/analgesic drug sales)	Cross-correlation, Graphical comparison	Low correlation Pearson r^2^ = 0.24 (p<0.005) Similar increases during the fall (norovirus) and influenza peak. Increase in ED GI visits during late winter (rotavirus), but no increase in drug sales.	-	Less sensitive than ED system	-	-
**Author**	**Syndrome**	**Reference data sources**	**Drugs selected**	**Statistical methods**	**Correlation strength**	**Correlation timeliness**	**Detection sensitivity**	**Detection specificity**	**Detection timeliness**
Edge et al. [16] 2004	GI	Emergency room visits for acute GI, number of GI cases from case series investigations (waterborne outbreak)	Saskatchewan: four commonly used antidiarrheals and antinauseants Ontario: 12 products (antidiarrheal, antinauseant, rehydration products)	Graphical comparison(Ontario, Saskatchewan), CUSUM, moving average (Ontario)	Trends of OTC products comparable to the outbreak epidemic curve (Saskatchewan,Ontario)	-	100% exceeded threshold during the outbreak period (Ontario)	100%	Not earlier
Magruder et al. [21] 2004	ILI	Outpatient insurance-claim diagnoses for acute respiratory conditions, from 13,000 clinics and doctors’ offices	Remedies for treating influenza (common use)	Cross-correlation	Seasonal trend: Pearson r (between 0.95 and 0.99)	1- 3 week lead	-	-	-
					Non-seasonal trend: Pearson r (between 0.25 and 0.75)	No repeatable lead time			
Hogan et al. [18] 2003	ILI and GI	Hospital-discharge diagnoses of respiratory and diarrheal disease in children (for all hospitals in Pennsylvania, in Utah, and 95% of Indiana).	Electrolyte products	Cross-correlation, EWMA	Pearson r = 0.90 (95% CI, 0.87-0.93)	Sales preceded diagnoses by 1.7 weeks (95% CI, 0.50-2.9)	100%	100%	Electrolyte sales preceded detection from diagnoses by an average of 2.4 weeks (95% CI, 0.1-4.8) Detection earlier in 12/18 outbreaks
Magruder [20] 2003	ILI	Outpatient insurance-claim diagnoses for acute respiratory conditions	Cold remedies: 622 products (then grouped in categories by an expert in pharmacoepidemiology)	Cross-correlation	Pearson r = 0.9	Mean lead times of 2.8 days	-	-	-
Davies et al. [15] 2003	ILI	Emergency admission data from Nottingham City Hospital NHS Trust.	Cold and flu remedies (cold, cough, decongestant, throat preparation)	Correlation, Peak comparison, Threshold detection method	National and local sales positively correlated with admissions in 98/99 and 99/00, not 00/01	-	100%(for local sales)	100% (for local sales)	Rate of local sales exceed threshold of 1000 units per week 2 weeks prior to peak in emergency admissions
**Author**	**Syndrome**	**Reference data sources**	**Drugs selected**	**Statistical methods**	**Correlation strength**	**Correlation timeliness**	**Detection sensitivity**	**Detection specificity**	**Detection timeliness**
Stirling et al. [26] 2001	GI	Telephone survey from a sample of households: number of persons with diarrheal symptoms and/or with stool specimen positive to *C. parvum* oocysts. (waterborne outbreak)	Common antidiarrheal (determined by each pharmacist)	Descriptive analysis	A fivefold increase in sales during the epidemic period	-	-	-	-
Proctor et al. [24] 1998	GI	Comparison with eight sources (laboratory confirmed cases of *Cryptosporidium*, clinically defined cases) (waterborne outbreak)	Antidiarrheal: Imodium, Pepto Bismol, Kaopectate	Descriptive analysis	Significant increase in drug sales during epidemic period	-	-	-	-
Rodman et al. [25] 1997	GI	Cases of cryptosporidiosis (5 waterborne outbreaks)	Antidiarrheal drugs	Descriptive analysis	Milkauwee: increased 20 fold; Las Vegas: no data; Collingwood: increased in 2 of 3 stores;Kelowna: increased 3 fold;Cranbrook: increased	-	-	-	-
Welliver et al. [29] 1979	ILI	Laboratory count of influenza B	Children’s aspirin, adult antipyretics, cold remedies	Determination of the% of sales increase, peak comparison	Sales of cold remedies averaged 185% above the baseline value during the peak influenza activity	-	-	-	-

*Abbreviations: ILI* Influenza-like illness, *GI* Gastrointestinal, *RSV* Respiratory syncytial virus, *ATC* Anatomical Therapeutic Chemical classification system, *CUSUM* Cumulative sum control chart, *OTC* Over-the-counter drugs, *EWMA* Exponentially Weighted Moving Average.

Reference data of the disease included medical case reports [20]-[22], diagnostic registries of microbiological laboratories [16],[24],[28],[29], hospital admission or discharge data [18]-[22],[27], or clinical emergency department data [14]-[16]. The selection of indicator drugs in these studies was based on the literature or expert opinion. For example, Edge et al. [17] selected all anti-nauseant and antidiarrheal OTC drugs for gastrointestinal surveillance. In Stirling et al. [26], pharmacists determined which common antidiarrheal drugs they would report.

Two methods were commonly used to compare drug-sale and diagnostic data time series: correlation analysis and signal detection comparison (Table 2). Ten studies used cross-correlation function to measure the similarity of two curves and to determine the time lag at which the correlation between the datasets is maximized. Cross-correlation is a standard method to determine the time delay between two signals. In three studies, only correlation between the time series was examined without analyzing time-lagged relationship. Six studies used aberration detection methods to evaluate whether and by how long the date of signal detection by drug sales precedes the signal based on diagnostic data. The signal definition for aberration detection was based on either a simple threshold to define alerts [15] or more complex algorithms such as the Serfling method [14], ARIMA models [19], the simple moving average method (MA), the cumulative sum method (CUSUM) [11],[16], or the exponentially weighted moving average (EWMA) [18]. These studies assessed the performance in terms of sensitivity, specificity and timeliness of disease outbreak detection. Five other studies [13],[24]-[26],[29] only evaluated whether drug sales showed a significant increase during a known epidemic period.

Twelve of 14 studies evaluating OTC sales retrospectively found significant correlations or a significant increase in drug sales [14]-[18],[20]-[22],[24]-[26],[29]. Only two studies didn’t found any consistent correlation. For example, Das et al. [14] found a poor correlation between OTC antidiarrheal drug sales and emergency department visits for diarrhea in New York City, with an r^2^ of 0.24. They found however an increase in sales during a known outbreak of norovirus. OTC drug sales preceded clinical data in three of eight studies that analyzed timeline correlations [18],[20],[21]. For example, in Hogan et al. [18], the correlation coefficient between electrolyte sales and hospital diagnoses of respiratory and diarrheal illness was 0.90 (95% CI, 0.87-0.93) when drug sales were assumed to precede clinical diagnosis data by 1.7 weeks. Outbreaks were detected with 100% sensitivity and specificity in 3 of 5 studies that analyzed signal detection [15],[16],[18]. Drug sales data provided an earlier outbreak signal in two of them [15],[18]. In Davies et al. [15], the rate of cough/cold sales exceeded a threshold of 1000 units per week two weeks before the peak in emergency department admissions during three consecutive winters. In Hogan et al. [18], detection from electrolytes sales occurred an average 2,4 weeks earlier than detection from hospital diagnoses of respiratory and diarrheal diseases.

Six of the seven studies that focused on prescribed drugs found strong correlations (r = 0.46-0.99) with clinical reference data or a significant increase in drug sales, without lead time however. The other study [11] showed that the CUSUM signal generated for prescriptions for macrolide antibiotics was linked to a pertussis outbreak in a county of New York State.

No association was observed between the type of reference data and the time lags observed.

#### Drug selection studies

An important challenge for drug-sales-based surveillance is identifying relevant indicator drug groups to monitor diseases. Five retrieved articles addressed this question. Characteristics of the studies are described in Table 3.

**Table 3:** **Methodology and results of drug selection studies included in a literature review of drug sales data analysis for surveillance of infectious diseases**

Author	Disease	Method	Results of the algorithm evaluation
Pelat et al. [33] 2010	GI	Hierarchical clustering procedure ,CUSUM	Identification of 4 therapeutic classes relevant to gastroenteritis outbreak detection. Detection performance of a multiple voter algorithm: sensibility 100%, specificity 95%, timeliness 1.7 weeks.
Cami et al. [31] 2009	ILI	Aggregate mining algorithm	Identification of product categories with outbreak detection performance superior to predefined categories and more strongly correlated with the disease data.
Wallstrom et al. [34] 2007	ILI	Unsupervised time-series clustering algorithm	Distinction between OTC products for allergy and OTC products for influenza symptoms
Li et al. [32] 2005	ILI/GI	Canonical correlation analysis	Identification of eight diagnoses that have strong association with electrolyte sales (r = 0.96)
Magruder et al. [21] 2004	ILI	Unsupervised stepwise clustering algorithm	Identification of 16 OTC product groups with similar historical trends

*Abbreviations: GI* Gastrointestinal, *CUSUM* Cumulative sum control chart, *ILI* Influenza-like illness, *OTC* Over-the-counter drugs.

Two studies [21],[34] developed methods to find homogeneous groups of OTC products. The authors used unsupervised clustering algorithms for aggregating OTC products in groups sharing similar sales histories. For example, Magruder et al. [21] first assigned OTC products for respiratory diseases to subgroups qualitatively based on indication, dose form, and age group. A stepwise hierarchical clustering algorithm was then used to form categories sharing a similar sales history, leading to a set of 16 product categories.

In two studies [31],[33], the authors developed procedures to identify the drugs correlating with disease incidence. Clusters were formed specifically for a particular disease. In Pelat et al. [33], a hierarchical clustering procedure was applied to the time series of all therapeutic classes and the acute diarrhea incidence rate reported by a network of general practitioners. Four therapeutic classes were found to cluster with diarrhea incidence and an algorithm based on the selected drugs allowed the detection of epidemics with a sensibility of 100%, a specificity of 95% and a timeliness of 1.7 weeks before official alerts.

#### Prediction studies

In three studies [35]-[37], the authors developed models to predict clinical data based on drug sales data.

Vergu et al. [37] used a Poisson regression model on selected OTC sales to forecast influenza-like illness (ILI) incidence as recorded by a sentinel network of general practitioners. The forecast at the national level 1-3 weeks ahead showed a strong correlation with observed ILI incidence (r = 0.85-0.96).

Najmi et al. [35] used least mean square filtering methods to estimate the incidence of emergency room consultations for respiratory conditions from past and present sales of groups of cold-remedy sales. In a later article [36], they succeeded in extending the estimation algorithm for predicting increases in clinical data several days in advance.

## Discussion

The evidence gathered in this systematic literature review suggests that drug sales data analysis can be a useful tool for surveillance of acute respiratory and gastrointestinal infections.

As could be expected, prescribed drug sales data were strongly correlated with clinical case reporting. No lead time was observed, which is consistent with the fact that patients purchase drugs after seeing a healthcare professional. Analysis of prescribed drug sales data may nevertheless have an additional utility for epidemic detection, as these data might be available with a shorter delay than clinical surveillance data [27].

A high correlation between OTC drug sales data and reference surveillance data were found in almost all the retrospective studies. Several studies also showed that OTC drug sales can serve as an early indicator of disease epidemics. Patients may buy nonprescription drugs during the early phase of illness when they become symptomatic, before consulting a health practitioner [38]. A surveillance system based on drug data should ideally detect all the outbreaks, rapidly, with a low false alert rate. However, few studies in the review determined the sensitivity and specificity of the outbreak detection and those aspects should be analyzed in more details in future studies.

Surveillance based on OTC drug sales could be particularly relevant for diseases whose prodromal phase persists for several days before the onset of more severe symptoms. For example, the early stages of dengue fever symptoms are nonspecific (fever, headache, myalgia, arthralgia, etc.) [39]. The occurrence of grouped cases could trigger an excess of nonspecific drug sales over baseline levels, which in turn could provide an early warning of outbreak in an endemic area.

Results from drug selection studies showed that it is possible to identify groups of products strongly associated with incidence data, which can then be used to predict future trends in clinical data and help public health authorities to prepare response planning. Such product selection procedures, however, depend on the existence of large clinical surveillance databases of the diseases concerned.

Similarly, the validity of drug sales data analysis has been evaluated mainly for two disease groups, respiratory and gastrointestinal illness, for which clinical reference data, used as the gold standard, are readily available. Pertussis and syphilis have been evaluated in only one study each, and still require further confirmation. The concept of drug-based surveillance therefore needs to be validated for other infectious diseases.

All the studies were conducted in developed countries or area. Surveillance based on drug sales data requires electronic information systems for routine data analysis. Besides, its implementation requires that the population has access to the health care system and mainly buy drugs in pharmacies. This could limit the use of drug based surveillance systems in developing countries.

By improving the timeliness of epidemic detection compared to clinical data and giving information from a larger part of the population, drug sales data can be an additional source of information for already monitored diseases. Besides, drug sales data analysis could have its greatest value in the surveillance of diseases for which clinical surveillance is cumbersome and costly, or where substantial under-reporting is suspected. To confirm the selected drug group as a valid proxy of disease, clinical surveillance may be conducted for a defined period in a representative population. Examples of diseases for which this would be useful are typically varicella, urinary infections, allergies/asthma, and parasitic diseases.

Ideally, the drugs to be monitored should be specific to the disease and widely used to treat it in order to maximize the sensitivity of the signal. For example, benzylpenicillin benzathine 2.4 MUI is the quasi exclusive treatment for syphilis infection [13] and is a good candidate. In contrast, the treatment of measles is mostly symptomatic without a specific drug, which makes this disease unattractive for this approach. Another limitation applies to diseases that are usually treated in hospitals or specialized centers, such as tuberculosis.

Surveillance based on drug sales, may not be appropriate to accurately estimate incidence of diseases, as the source population size is not precisely known. Moreover, it may be difficult to link the number of drug packages sold to the number of patients with disease. However, the method is very efficient to determine temporal dynamics of a situation and to detect abnormal phenomena. Surveillance based on drug sales is therefore well adapted to diseases with seasonal variations such as norovirus gastroenteritis, influenza and other infectious respiratory agents, or community outbreaks (foodborne illnesses, waterborne illnesses, hepatitis A, etc.).

Drug sales can be influenced by store promotions, sales period (holidays, weekends), and the media. Also, we do not know whether people buy medications to treat a disease they currently have or a disease they fear they may have in the near future. For example, during the media coverage of avian influenza A (H5N1) in the US, an increase in antiviral medications sales was observed [40], which corresponded to stockpiling behavior of the population.

Health-seeking behaviors also vary by demographic, social, cultural, and economic factors. A survey [41] in Canada analyzed the healthcare-seeking behaviors of 351 patients with acute gastroenteritis. They found significant differences (patient age and sex) between the patients who used OTC drugs and those who did not. Consequently, factors that prompt self-medication should also be taken into account. The usefulness of drug sales based surveillance is also dependent on the available resources and the organization of the health care system. OTC drug sales surveillance is for example less relevant in countries where reimbursement rate are high and patients mainly get prescribed drugs.

Population mobility, particularly in tourist areas, may lead to an increase in remedy sales, which could wrongly be interpreted as a disease outbreak. Inversely, patients with high geographical mobility may not be included in the region of study and lead to an underestimation of the magnitude of an epidemic.

Despite some limitations, routine collection and analysis of drug sales data are likely to be developed in the coming years. Many automated surveillance systems that collect drug data the day after the sales have been implemented in the last decade [14],[19],[20],[27]. They allow a rapid assessment of the public health situation. Early detection of outbreaks allows public health authorities to set up epidemic investigations and control measures sooner. Most studies included in this review were published after the year 2000, with their number increasing recently. They illustrate the need for improved surveillance systems, evidenced by recent public health crises (e.g., anthrax in 2001, the SARS outbreak in 2003, the A/H1N1 influenza pandemic in 2009, etc.). Drug sales data present indeed many advantages in terms of public health surveillance. Data can be obtained in a real-time manner and usually cover a large portion of the population. Data collection may be exhaustive, without selection of specific sales, and allows the simultaneous monitoring of a large number of diseases, especially new or emerging diseases.

Although non-specific, drug sales data are directly linked to patients’ health conditions. Drug sales data are therefore more specific than other syndromic surveillance data, such as tracking search patterns on the web and are likely to reflect more accurately disease activity in the population. Moreover, it should be noted that alternative sources of data for disease surveillance are currently under development. Healthcare management databases that can provide exhaustive information on drug consumption and diagnosis, as the Dossier Médical Personnel [42] in France, are promising tools for disease surveillance.

Our review may be affected by a publication bias since studies unable to show correlations between drug sales and reference data may have been less published. In addition, selections bias may have occurred in the studies. Indeed, some studies in the review were based on a limited number of pharmacies and/or a limited study period (e.g. less than one year). Language bias may exist as we were not able to identify studies published in languages other than English and French. The review focused on the temporal dynamics of infectious disease; consequently, further analyses are required to determine the capacity of these systems to efficiently monitor other aspects of infectious diseases such as spatial spreading.

## Conclusion

This review suggests that the analysis of drug sales data is a promising method for surveillance and outbreak detection of infectious diseases. It has the potential to trigger an outbreak alert earlier than most surveillance systems. However, the main challenges consist in the appropriate selection of indicator drug groups and the validation of this approach for diseases for which no or poor quality clinical surveillance data exists. The usefulness of the approach also depends on the available resources and the organization of the health care system. Drug sales databases with real-time or near real-time data transmission are available in several countries; future studies should be encouraged to expand their use on other infectious diseases.

## Authors’ contributions

All authors contributed to the study’s design. MP and JM carried out the literature search and reviewed articles. MP drafted the manuscript. All authors interpreted the results, revised and approved the final manuscript.

## Authors’ information

Mathilde Pivette has a Doctor of Pharmacy degree and a Master of Public Health. She is a PhD candidate in epidemiology at the French School of Public Health. Her research interest lies in the analysis of drug sales data for disease surveillance.

## Authors’ original submitted files for images

Below are the links to the authors’ original submitted files for images. Authors’ original file for figure 1
