# Supplementary figures and images for: Drug sales data analysis for outbreak detection of infectious diseases: a systematic literature review
Source: BMC Infect Dis. 2014 Nov 18;14:604. doi: 10.1186/s12879-014-0604-2 (PMC4240820; doi:10.1186/s12879-014-0604-2)

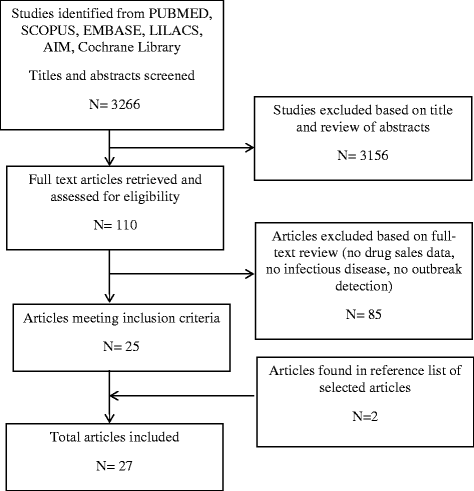

Supplement: Supplementary file 1 — Authors’ original file for figure 1 [file 12879_2014_604_MOESM1_ESM.gif]
